# Paired personal interaction reveals objective differences between pushing and holding isometric muscle action

**DOI:** 10.1371/journal.pone.0238331

**Published:** 2021-05-06

**Authors:** Laura V. Schaefer, Frank N. Bittmann

**Affiliations:** Division Regulative Physiology and Prevention, Department Sports and Health Sciences, University of Potsdam, Potsdam, Germany; Universidade Estadual Paulista Julio de Mesquita Filho - Campus de Bauru, BRAZIL

## Abstract

In sports and movement sciences isometric muscle function is usually measured by pushing against a stable resistance. However, subjectively one can hold or push isometrically. Several investigations suggest a distinction of those forms. The aim of this study was to investigate whether these two forms of isometric muscle action can be distinguished by objective parameters in an interpersonal setting. 20 subjects were grouped in 10 same sex pairs, in which one partner should perform the pushing isometric muscle action (PIMA) and the other partner executed the holding isometric muscle action (HIMA). The partners had contact at the distal forearms via an interface, which included a strain gauge and an acceleration sensor. The mechanical oscillations of the triceps brachii (MMGtri) muscle, its tendon (MTGtri) and the abdominal muscle (MMGobl) were recorded by a piezoelectric-sensor-based measurement system. Each partner performed three 15s (80% MVIC) and two fatiguing trials (90% MVIC) during PIMA and HIMA, respectively. Parameters to compare PIMA and HIMA were the mean frequency, the normalized mean amplitude, the amplitude variation, the power in the frequency range of 8 to 15 Hz, a special power-frequency ratio and the number of task failures during HIMA or PIMA (partner who quit the task). A “HIMA failure” occurred in 85% of trials (*p* < 0.001). No significant differences between PIMA and HIMA were found for the mean frequency and normalized amplitude. The MMGobl showed significantly higher values of amplitude variation (15s: *p =* 0.013; fatiguing: *p* = 0.007) and of power-frequency-ratio (15s: *p* = 0.040; fatiguing: *p* = 0.002) during HIMA and a higher power in the range of 8 to 15 Hz during PIMA (15s: *p* = 0.001; fatiguing: *p* = 0.011). MMGtri and MTGtri showed no significant differences. Based on the findings it is suggested that a holding and a pushing isometric muscle action can be distinguished objectively, whereby a more complex neural control is assumed for HIMA.

## Introduction

In sports and movement science, isometric muscle action is usually measured by pushing against a stable resistance. However, subjectively one can perform it by pushing against a stable resistance or by resisting an object in a holding manner. For example, during arm wrestling, one can pursue the strategy to push against the partner until he or she gives in or one can try to hold as long as possible until the partner cannot push anymore. In both cases, no motion occurs as long as both partners are able to maintain the reaction force, thus, the muscle length and joint angle stay stable. In a recent study, we suggested that those forms are referred to as the pushing isometric muscle action (PIMA) and the holding isometric muscle action (HIMA), respectively [1]. Until now, the proposed two forms of isometric muscle action are rarely the objective of movement or sport science research. Garner et al. hypothesized that two forms of isometric muscle action exist [2]. They rejected the hypothesis since no differences in the amplitude or frequency of electromyography (EMG) of the soleus and gastrocnemius muscles appeared during the performance of HIMA and PIMA. Previous studies by Enoka’s research group [3–7] investigated the assumed different forms and denoted them position task (HIMA) or force task (PIMA), respectively. Thereby, the position task (HIMA) was performed by holding an inertial load and maintaining a constant position of the limb, which was freely movable. During the force task (PIMA) a force level (same intensity as during the position task) should be maintained by pushing against a stable resistance. The main finding was that the time to task failure was significantly longer during the force task compared to the position task for the elbow flexor muscles at intensities of 15% of the maximal voluntary isometric contraction (MVIC) [4,5,7]. However, those results were only found at low intensities (15% to 30% of the MVIC) and with the forearm positioned horizontally. With higher force intensities of 45% and 60% of the MVIC, no differences between HIMA and PIMA were observed [5]. No differences in time to task failure were found at any intensities having the forearm positioned vertically [4]. The averaged EMG at exhaustion as well as the power in the frequency range of 10 to 29 Hz were significantly higher during the force task compared to the position task. The mean amplitude and mean frequency of EMG showed no significant differences between both tasks [5].

The recently published study of our laboratory [1] partly supported those results, partly disagreed with them. The setting included the recordings of mechanomyography (MMG) of the triceps brachii and the abdominal external oblique muscles as well as of mechanotendography (MTG) of the triceps brachii tendon, whereas no EMG was captured. The results revealed a significantly longer time to task failure at an intensity of 80% of the MVIC with vertically positioned forearm during PIMA compared to HIMA. The MMG of the triceps brachii in the last 10% of measurement time (exhaustion) showed significantly higher amplitudes during PIMA vs. HIMA. The mean frequency did not differ significantly between both tasks. However, the MTG of triceps tendon showed a significantly higher power in the frequency ranges of 10 to 29 Hz and 8 to 15 Hz, respectively, during HIMA compared to PIMA [1]. This is opposite to the findings of Hunter et al. [7], who found a higher power during PIMA. The differences might be attributed to the different settings and, of course, different methods (EMG vs. MMG).

Using MMG the mechanical muscle oscillations are recorded. It can be interpreted as the mechanical counterpart to EMG [8]. The signal to noise ratio of MMG raw data is excellent using piezoelectric sensors [9]. Furthermore, the preparation of skin is less complex and, therefore, the application is easier and less error prone. Therefore, the use of MMG is a promising tool to get insights into the neuromuscular system.

Based on the above-mentioned findings it is assumed that two different forms of isometric muscle action can be distinguished by objective parameters. It is not that easy to enable especially the holding isometric muscle action during measurements. In the former performed investigation, a pneumatic device was used so that the participant could realize the holding function [1]. Thereby, the push rod of the pneumatic system pushed against the forearm of the subject. Another possible approach is that two persons interact mutually. Thereby, one partner pushes against the other one (PIMA), while the second partner reacts to the force and resists by holding isometrically (HIMA). This setting is assumed to be even more challenging, since two neuromuscular systems interact. It has already been shown that thereby the MMG and MTG signals are able to develop coherent behavior in the sense of synchrony not only within one subject, but also between the muscles and tendons of both partners [9,10]. Using MMG, the mechanical micro-oscillations of muscles, which appear in frequency ranges around 10 Hz, are captured as one form of motor output [10,11]. This enables also to draw conclusions about central processes [12]. By maintaining a given force level, central structures must be activated to control the muscle action. Those motor control and regulating processes are assumed to get even more challenging if the neuromuscular system has to react to exteroceptions. This is rather the case for HIMA than for PIMA.

The aim of the present study was to investigate whether or not the two forms of isometric muscle action can be distinguished by quantifiable parameters also during an interpersonal motor task. Based on the previous results, it was hypothesized that the partner who performs HIMA will fail to maintain the task during the interaction. Furthermore, it was assumed that there will be no differences between HIMA and PIMA for the mean frequency and amplitude of the MMG and MTG signals but for the amplitude variation and power distribution. The present investigation might provide further insights into isometric muscle function and should support the research concerning the suggested two forms of isometric muscle action.

## Methods

### Participants

In total, 20 healthy athletic subjects (*n* = 10 male, *n* = 10 female) volunteered to participate in the study, which were assigned to ten same sex couples for the measurements. The participants were sport students at the University of Potsdam (Germany) collected from the Bachelor studies of sports for teaching profession or of sports therapy and prevention. The average age, weight and height of the male participants were 22.1 ± 2.4 years, 75.2 ± 6.9 kg and 181.5 ± 5.1 cm and for the female participants 21.6 ± 2.1 years, 60.4 ± 3.5 kg and 168.3 ± 4.4 cm. Exclusion criteria were complaints of the upper extremities, shoulder girdle and spine within the las six months prior to measurements or any other health issue. The participants were asked to avoid sport activity in the 24 hours before measurements to avoid fatiguing effects.

The study was approved by the local ethics committee at the University of Potsdam (Germany), approval no. 64/2016, and was conducted according to the Declaration of Helsinki. All participants were informed in detail and gave written informed consent to participate.

### Setting

**Fig 1:**
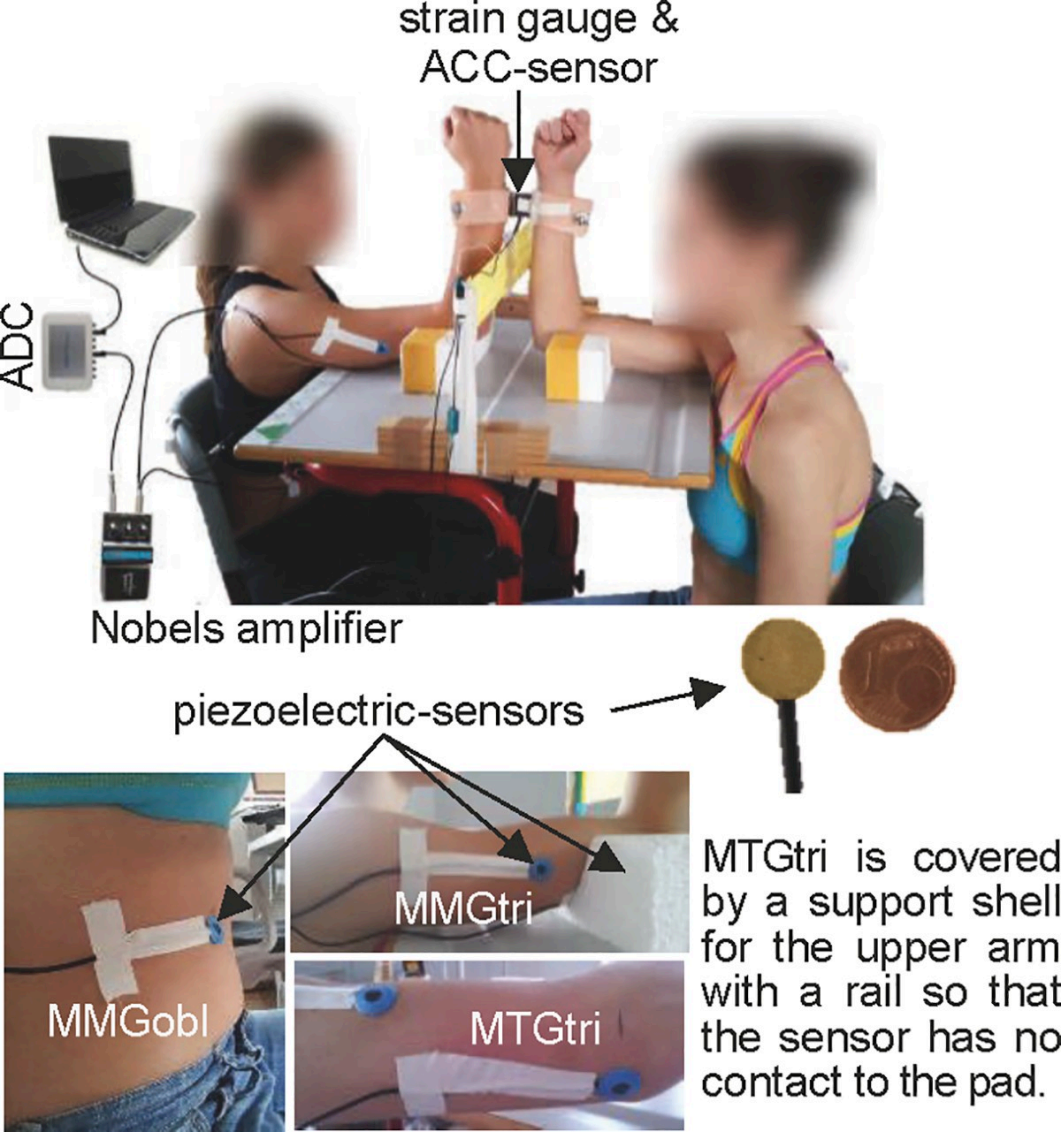
Setting. The partners are interacting with their distal forearms, which are coupled by two shells incl. the strain gauge and accelerometer. The piezoelectric sensors for MMG are fixed on the abdominal external oblique muscle (MMGobl), on the lateral head of the triceps brachii muscle (MMGtri) and–to capture the MTG of the triceps tendon (MTGtri)–on the olecranon fossa. The MMG/MTG-signals are amplified with the Nobels pre-amp booster pre-1. (Fig 1 of [9]; modified with permission).

The setting was identical to the investigations reported in Schaefer & Bittmann [9] and Schaefer et al. [10] (Fig 1): The participants were sitting opposite, so that the measured dominant vertically positioned forearms were located in one plane. The angles between leg and trunk, arm and trunk as well as the elbow angle measured 90°. An interface with two shells of a thermic deformable polymer material (orthopedic material) was positioned proximal of the ulnar styloid processes of both participants and, thereby, connected the partners. To record the reaction force between the participants, a strain gauge was fixed between the shells (model: ML MZ 2000 N 36, modified by biovision). Furthermore, a one-axial acceleration sensor (sensitivity: 312 mV/g, range ± 2 g, linearity: ± 0.2%; comp. biovision) was positioned on the strain gauge between the shells to capture the accelerations along the longitudinal acting force vector during the measurements. To record the mechanical muscular and tendinous oscillations (MMG/MTG) piezoelectric sensors (Shadow SH 4001) were fixed by ECG-tape on the skin above the bellies of the lateral head of the triceps brachii muscle (MMGtri) and its tendon (MTGtri) as well as on the ipsilateral abdominal external oblique muscle (MMGobl; 2cm medial of the free end of the 11^th^ rib). The triceps brachii muscle and its tendon were chosen since the main isometric muscle action in this position was performed by the elbow extensors. The ipsilateral abdominal external oblique muscle served as an important core stabilizer within the kinematic chain and, therefore, was also recorded. The MMG-/MTG-signals were amplified by Nobels preamp booster pre-1. All signals were conducted to an A/D-converter (14-bit, National Instruments) and were recorded by the software NI DIAdem 10.2 (National Instruments) on a measurement notebook (Sony Vaio: PCG-61111M, Windows 7). The sampling rate was set at 1000 Hz. Between both subjects a “border” was constructed using a Thera band®. The height of the upper edge of the Thera band was 20 cm. The distance between the edge and the forearm of each participant was ~2.5 cm. Therefore, if the forearm of one participant touched the band, its elbow angle was extended by 7°, thus, the partner had yielded by 7°. This was assessed as considerably leaving the isometric position and led to the abortion of the measurement. This range was accepted since the partners developed significant mutual oscillations at the coupling point.

### Procedure and measurement tasks

The measurements were performed in one session of approximately 60 minutes. No prior additional appointment for familiarization with the tasks took place.

The participants of each same sex pair should maintain an isometric muscle action using the elbow extensors at a reaction force of 80% of the MVIC (15s-trials) or 90% of the MVIC (fatiguing trials), respectively, of the weaker subject. The MVIC was recorded prior to the pair trials. In order to do this, each participant pushed singly in the measurement position against a shell of a fixed strain gauge and should reach its MVIC within 3s as well as should maintain it for 1s (2 trials). The position of the strain gauge was marked and the arm length was measured. The higher of both MVIC values of the weaker subject was used to calculate the force level of the pair trials.

Subsequently, two different measurement series were performed in the HIMA-PIMA-setting: (1) 15s-trials, whereby the isometric position should be sustained for 15s and (2) fatiguing trials, whereby the isometric position should be maintained as long as possible. The abortion criteria were either the contact to the border of one subject or a force decrease, whereby one or both partners stopped the interaction due to exhaustion. In total, six 15s-trials were performed at 80% of the MVIC of the weaker person. In the first three of those six trials participant A performed PIMA and B performed HIMA. For the other three trials, the tasks changed (A: HIMA; B: PIMA). The resting period between the trials was 60s. Afterwards, four fatiguing trials were performed at 90% of the MVIC. The higher intensity of 90% of the MVIC was chosen to limit the measurement duration. Again, the setting was complementary (PIMA vs. HIMA) with a change of the allocated task after the first two trials. The tasks for the participants to start with (HIMA or PIMA) were randomized for both measuring series. The resting period between the trials was 120s.

As mentioned above, each partner had different tasks during the pair trials, which changed within the measurement series: participant A pushed isometrically against participant B (PIMA), while participant B resisted the force applied by participant A isometrically in a holding manner (HIMA). Basically, PIMA is understood as an isometric muscle action in direction of elbow extension, like a kind of “stopped concentric action” without a change of joint angle. HIMA is seen as isometric reaction to an external force (here the force of the partner), which works in direction of elbow flexion. Thus, HIMA can be understood as a kind of “stopped eccentric action”. A more detailed description of HIMA and PIMA can be found in Schaefer & Bittmann [1]. In the present setting, the holding participant should behave like a “wall” to hinder the partner to extend the arm, thus, maintaining the position ideally completely stable. In order to complete this task, the holding subject had to adapt to the applied force of the pushing partner. Therefore, the HIMA task required a high level of kinesthetic adaptation. The pushing partner controlled the force intensity via a dial instrument showed on a monitor in real time (biofeedback). Thus, the pushing partner had a visual feedback and should initiate the force. The isometric position can only be sustained if both partners maintain the given force level. If one partner contacted the border (Thera band®) between the pair, the measurement was stopped. Thereby, one partner flexed and the other partner extended the elbow joint with an amount of 7°. Due to the arising mutual oscillations the accepted amount of 7° was necessary. The pushing partner would contact the border in case the holding counterpart would get tired first. Due to the task of the holding subject (just reacting to the force of the pushing partner), normally this subject should not be able to contact the border during the interaction. In case the pushing subject would get tired first, the holding partner should not extend his/her elbow angle, because he/she was instructed to stay in a stable position. In a case like this, the force level would decrease. Since this interaction is not trivial, the holding subject sometimes followed the yielding of the pushing partner in order to maintain the force level. Thereby, the initially pushing partner gave in and the formerly holding partner extended the elbow angle. This was the case in two trials of one single subject (see results and a critical discussion in the limitation section).

### Data processing

The main objective concerning the two forms of isometric muscle action is the endurance capability. In the present setting this was assessed qualitatively by counting the task failures. In case the PIMA partner contacted the border or the HIMA partner quit the task at first, it was rated as “HIMA failure”. If the PIMA partner quit the task accompanied by a force break down, it was designated as “PIMA failure”. As mentioned above, one subject contacted the border during HIMA. This was categorized as “PIMA failure”, too. If both partners stopped the interaction simultaneously, the interaction was classified as even. Nominal data were obtained (0 = “PIMA failure”, 1 = “HIMA failure”, 2 = “even”) to compare the task failures between HIMA and PIMA.

Considering the oscillatory signals, the isometric plateau was cut from all signals (MMG, MTG) using the force signal. For that, the isometric plateau was defined as the duration, in which the reaction force between both partners was maintained. Slipping parts were excluded and in that case, the longer isometric plateau was used for further consideration. The signals of the isometric plateau were filtered using a low pass Butterworth filter, (filter degree 5, cut-off frequency 20 Hz). Afterwards, the following parameters were considered: 1.) the mean frequency, 2.) the normalized mean amplitude, 3.) the normalized amplitude variation, 4.) the power in the frequency range of 8 to 15 Hz and 5.) a special power-frequency ratio (MQ_REL_), which was recently used in another investigation concerning patients with Parkinson´s syndrome [8].

1. The mean frequency of the MMG-/MTG-signals was calculated using a Python script, which computed the average of the time intervals between two consecutive amplitude maxima and calculated the frequency thereof. According to Pikovsky et al. [13], this method is applicable for chaotic signals as neuromuscular ones.
2. The normalized mean amplitude of the MMG-/MTG-signals was evaluated in Excel (IBM Microsoft Office) by calculating the arithmetic mean of the amplitude maxima normalized to the maximal amplitude of one signal.
3. The normalized amplitude variation of the MMG-/MTG-signals was evaluated in Excel (IBM Microsoft Office). The differences between two consecutive maxima each were calculated and averaged over all time points of one signal. This mean variation value was normalized to the arithmetic mean of amplitude maxima of the signal.
4. The power spectral density (PSD) of the MMG-/MTG-signal was calculated in NI DIAdem 10.2. The values of power in the frequency range of 8 to 15 Hz were transferred to Excel, where the arithmetic mean of the power was calculated per signal.
5. For the specific power frequency ratio (MQ_REL_) the power spectral densities (PSD) of the MMG-/MTG-signals were calculated in NI DIAdem. Afterwards, the ratio of the power in the frequency range of 3 to 7 Hz to the sum of power in the frequency range of 3 to 7 and 7 to 12 Hz was calculated in Excel (IBM Microsoft Office) for each signal using the equation:

$$MQ_{REL}=\frac{M\;of\;power\;in\;the\;frequency\;range\;of\;3\;to\;7\;\mathrm{Hz}}{\left(M\;of\;power\;in\;frequency\;range\;of\;3\;to\;7\;\mathrm{Hz})+(M\;of\;power\;in\;frequency\;range\;of\;7\;to\;12\;\mathrm{Hz}\right)}$$ (1)

The arithmetic mean (M), standard deviation (SD) and coefficient of variation (CV) of all parameters (1) to (5) were calculated per participant, measurement series (15s-/fatiguing-trials) and tasks (HIMA vs. PIMA). The signals of one MMGtri, the MTGtri of one pair and one MMGobl signal were defective and, therefore, eliminated from the statistics.

Group statistics. IBM SPSS Statistics 26 was used for executing the group comparisons concerning the parameters (1) to (5) to investigate the differences between the motor tasks HIMA and PIMA. The significance level was set at *α* = 0.05. Firstly, the data were tested regarding their normal distribution by the Shapiro-Wilk-test. If the normal distribution was fulfilled, the paired t-test was utilized. The effect size was calculated by Pearson’s r = $\left|\sqrt{\frac{t^{2}}{t^{2}+df}}\right|.$ In case of non-parametric data, the comparison between HIMA and PIMA was done by the Wilcoxon-test for paired samples. The effect size was calculated by Pearson’s r = $|\frac{z}{\sqrt{n}}|.$ For comparing the nominal data (“HIMA failure” vs. “PIMA failure” vs. “even”) between HIMA and PIMA a χ^2^-test was performed in Excel (IBM Microsoft Office), the effect size was calculated by phi Φ = $\sqrt{\frac{\chi^{2}}{N}}$.

## Results

**Fig 2:**
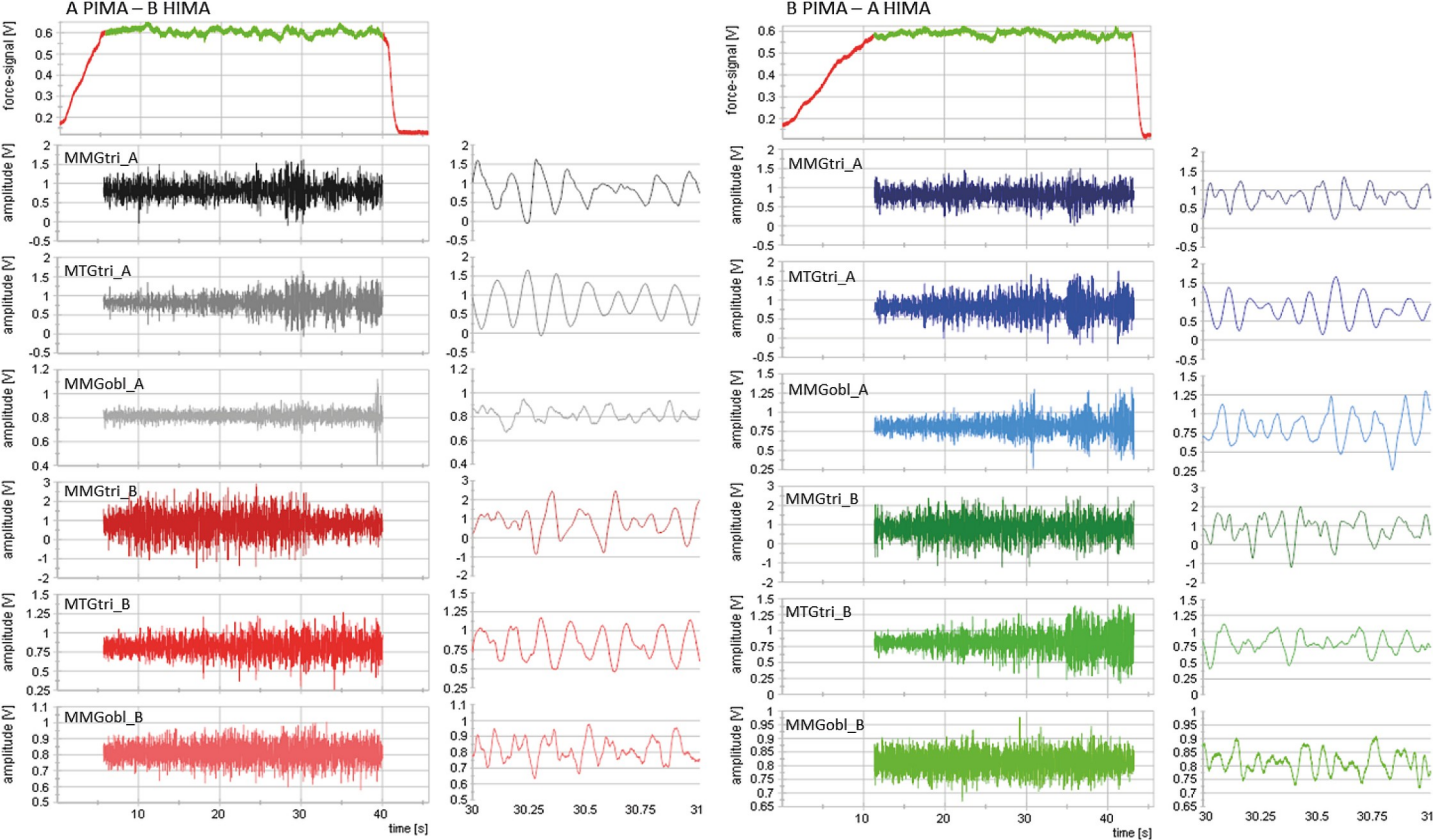
Exemplary raw signals. Displayed are the raw signals of two fatiguing measurements at 90% of the MVIC of one pair (A and B) of the strain gauge (V9 and the mechanomyo- and -tendographic signals (V) of the triceps brachii and the abdominal external oblique muscles and of the triceps tendon, respectively. Left diagrams: A PIMA, B HIMA; right diagrams: A HIMA, B PIMA. The 1s-intervals to the right of each diagrams of the whole duration illustrate the good signal quality of the oscillatory raw signals.

Fig 2 displays exemplary raw signals of two fatiguing measurements of one pair at 90% of the MVIC during HIMA vs. PIMA. Table 1 gives an overview of the group arithmetic means and standard deviations (M ± SD) of the MMG and MTG parameters.

**Table 1:** Overview of the MMG/MTG parameters.

sensor	trial	task	Normalized amplitude	Amplitude variation (%)	Mean frequency (Hz)	Power in frequency range of 8 to 15 Hz (V^2^/Hz)	MQ_REL_
**MMGtri**	**15s**	PIMA	0.323 ± 0.083	76.014 ± 17.121	13.737 ± 1.165	0.026 ± 0.040	0.262 ± 0.149
		HIMA	0.330 ± 0.089	76.583 ± 14.923	13.598 ± 1.220	0.029 ± 0.059	0.278 ± 0.137
	**fatiguing**	PIMA	0.352 ± 0.107	71.157 ± 15.567	13.569 ± 1.120	0.039 ± 0.049	0.225 ± 0.111
		HIMA	0.330 ± 0.075	73.239 ± 15.320	13.234 ± 1.218	0.035 ± 0.041	0.248 ± 0.134
**MTGtri**	**15s**	PIMA	0.296 ± 0.056	76.606 ± 17.964	12.473 ± 1.037	0.015 ± 0.022	0.307 ± 0.175
		HIMA	0.287 ± 0.057	82.324 ± 17.694	12.479 ± 1.578	0.010 ± 0.015	0.348 ± 0.149
	**fatiguing**	PIMA	0.291 ± 0.067	71.896 ± 19.307	12.377 ± 1.149	0.016 ± 0.020	0.283 ± 0.158
		HIMA	0.307 ± 0.061	70.645 ± 17.220	11.692 ± 1.346	0.011 ± 0.011	0.267 ± 0.179
**MMGobl**	**15s**	PIMA	0.297 ± 0.039	**73.334 ± 13.614**	14.097 ± 1.443	**0.002 ± 0.003**	**0.247 ± 0.163**
		HIMA	0.276 ± 0.047	**83.455 ± 14.946**	13.929 ± 1.273	**0.001 ± 0.002**	**0.308 ± 0.180**
	**fatiguing**	PIMA	0.298 ± 0.050	**70.372 ± 12.160**	13.697 ± 1.025	**0.003 ± 0.004**	**0.167 ± 0.099**
		HIMA	0.266 ± 0.074	**78.537 ± 15.677**	13.906 ± 1.462	**0.002 ± 0.003**	**0.249 ± 0.146**

Displayed are the arithmetic means and standard deviations (M ± SD) of the parameters concerning MMG/MTG of the whole group of n = 20 participants sorted by sensor, trial and task. Significant differences between HIMA and PIMA are written in bold (p < 0.05).

### MVIC and force levels

The MVIC amounted averagely 51.19 ± 22.45 Nm for the male participants and 25.83 ± 0.07 Nm for the females. The averaged difference of MVIC values between the partners of one couple was 15.08 ± 9.40 Nm (24.56 ± 14.49%, range: 0.53 to 40.58%) for males and 3.53 ± 3.19 Nm (13.03 ± 11.09%, range: 1.2 to 27.33%) for females. Therefore, the adjusted force intensity of 80% of the MVIC of the weaker partner amounted averagely 62 ± 10% of the MVIC of the stronger partner for males; for the females it amounted 70 ± 9% of the MVIC of the stronger partner.

During the 15s-trials at 80% of the MVIC, the force level amounted averagely 23.08 ± 7.07 Nm during PIMA of subjects A (= HIMA of subjects B) and 23.12 ± 7.00 Nm during PIMA of subject B (= HIMA of subject A) (*W* = -0.563, *p* = 0.574). During the fatiguing trials at 90% of the MVIC, the force level amounted averagely 26.17 ± 8.08 Nm during PIMA of subjects A (= HIMA of subjects B) and 26.28 ± 7.90 Nm during PIMA of subject B (= HIMA of subject A) (*W* = 0.327, *p* = 0.744). Due to the non-significant differences between the tasks HIMA und PIMA, the consideration of the following other parameters is based on a similar force level.

### Capability of maintaining the tasks

**Fig 3:**
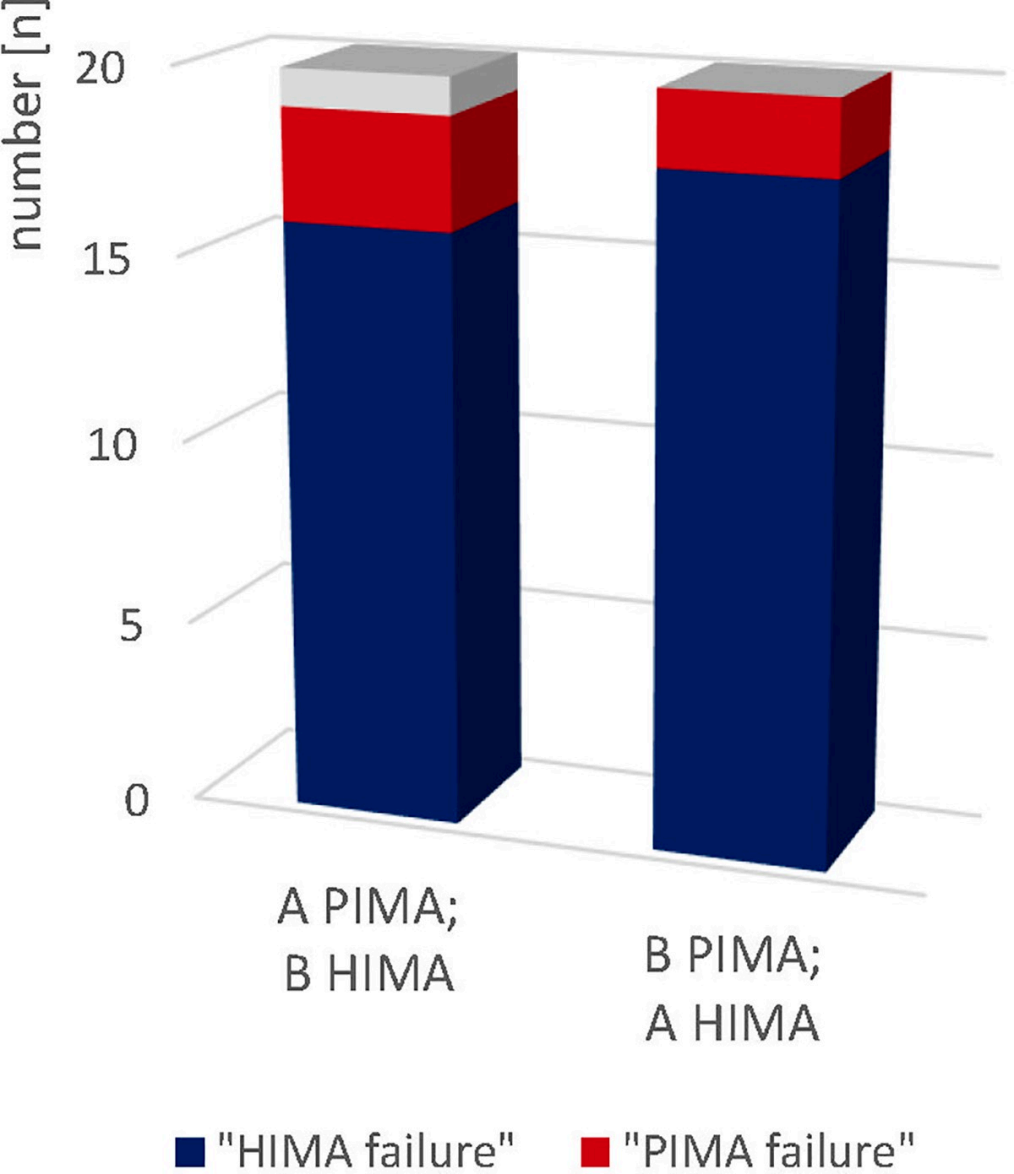
HIMA vs. PIMA failures. Displayed are the number of HIMA (n = 34, blue) and PIMA (n = 5, red) failures as well as the even (n = 1, grey) voted interaction during the 40 fatiguing trials grouped into subject A performs PIMA (B HIMA) vs. subject B performs PIMA (A HIMA). The tasks failures differ significantly between HIMA and PIMA (p < 0.001).

The mean duration time of the fatiguing interactions over all trials was 24.58 ± 11.44s. Since in all interactions one partner performed PIMA and the other one performed HIMA, the time to task failure is not suitable to evaluate possible differences between HIMA and PIMA. That is why the endurance capability is captured qualitatively by counting the number of failures of each subject during the fatiguing trials. A “HIMA failure” occurred in 34 out of 40 trials (85%), whereas five PIMA failures arose (5 of 40; 12,5%). During one trial, the interaction was assessed as even since both partners stopped the interaction simultaneously because of exhaustion (2.5%) (Fig 3). The Chi-square test revealed a significant difference between PIMA and HIMA (*χ^2^*(2) = 25.87, *p* < 0.001) with a strong effect of Φ = 0.75.

### Amplitude and amplitude variation

The normalized amplitude showed no significant difference between HIMA and PIMA for MMGtri, MTGtri and MMGobl during the 15s and fatiguing trials (Table 1). However, for MMGobl the comparison is just not significant, whereby during PIMA the normalized amplitude tends to have higher mean values compared to HIMA (*t*(19) = 2.048, *p* = 0.055, *r* = 0.42) (Table 1; M and SD of each participant can be found in supplementary material).

**Fig 4:**
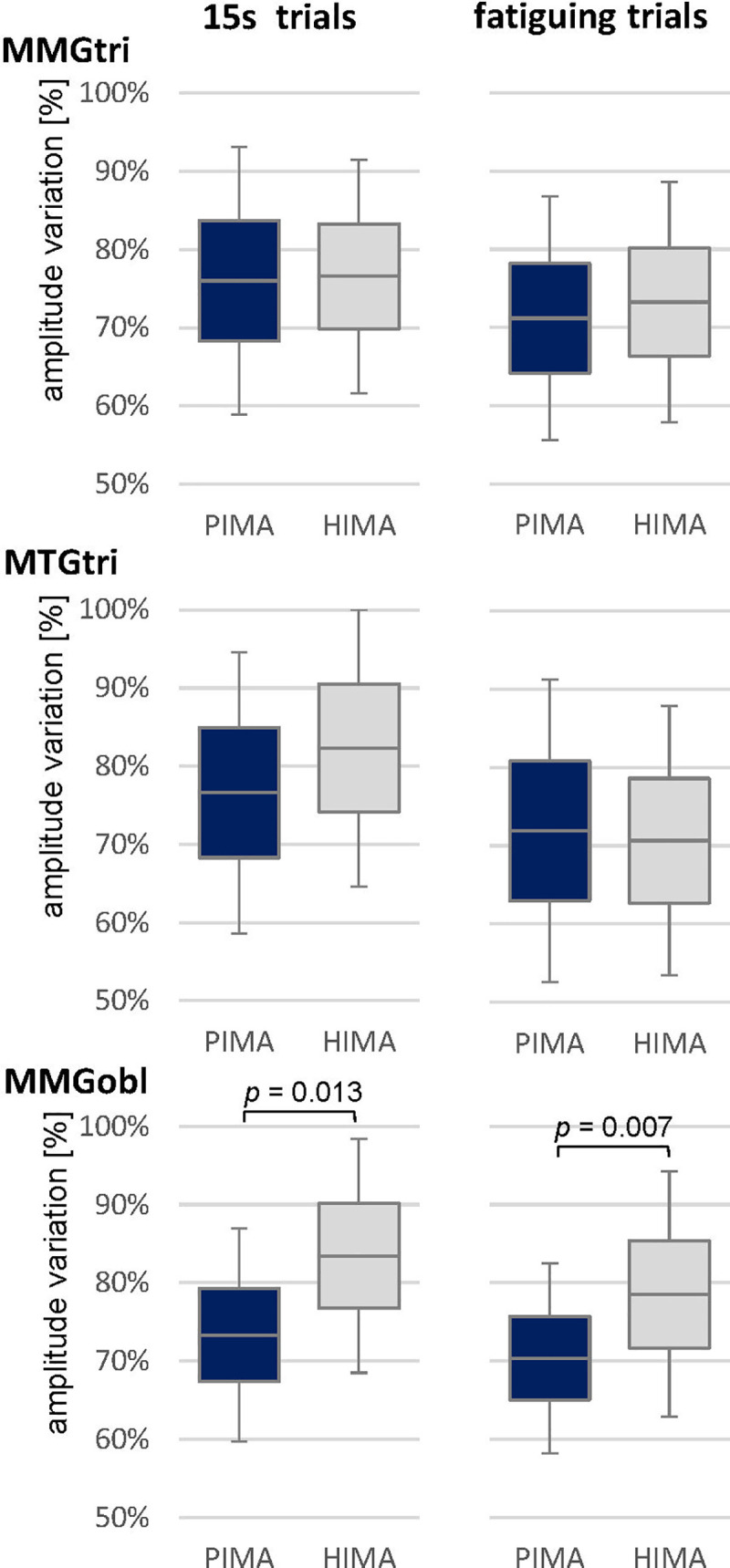
Amplitude variation. Displayed are the arithmetic means, standard deviations (error bars) and the 95%-confidence intervals of the amplitude variation (%) of the signals MMGtri, MTGtri and MMGobl during the 15s trials (left) and fatiguing trials (right) comparing PIMA (blue) vs. HIMA (grey). T-test was significant for MMGobl, p-values are given in case of significance.

The arithmetic means of variation of amplitude maxima revealed no significant difference between PIMA and HIMA for MMGtri and MTGtri, but they differed significantly for the MMGobl in the 15s-trials (t(19) = -2.759, *p* = 0.013, *r* = 0.55) and in the fatiguing trials (t(19) = -3.038, *p* = 0.007, *r* = 0.57) (Table 2, Fig 4). Thereby, the amplitude variation was significantly higher during HIMA compared to PIMA. Looking at the single cases, 15 of 20 participants showed a higher amplitude variation during HIMA, although the couples did not change, only the tasks HIMA and PIMA.

**Table 2:** Amplitude variation.

		MMGtri				MTGtri				MMGobl			
		15s		fatiguing		15s		fatiguing		15s		fatiguing	
Couples		PIMA	HIMA	PIMA	HIMA	PIMA	HIMA	PIMA	HIMA	PIMA	HIMA	PIMA	HIMA
**1**	1	53.7±12.9	68.9±6.8	47.6±10.7	60.4±9.6	49.7±2.7	81.9±11.9	62.7±1.9	67.6±11.9	54.3±13.7	64.5±12.6	72.1±7.0	67.2±20.0
	2	61.8±7.8	80.1±11.1	80.1±11.1	64.9±8.6	47.4±6.4	69.3±4.3	47.4±4.6	57.6±6.4	72.6±4.0	78.7±12.7	68.3±6.7	59.2±12.3
**2**	3	113.8±6.4	113.7±8.4	113.7±8.4	77.1±1.7	85.7±12.7	98.2±20.1	54.0±0.9	57.2±0.6	95.8±3.4	92.9±7.3	84±14.6	100.5±11.9
	4	91.9±10.0	85.8±10.7	85.8±10.7	83.6±2.9	96.1±14.0	108.2±11.2	96.6±13.7	67.4±13.5	97.1±3.0	88.6±7.2	86.2±4.8	90.0±3.9
**3**	5	73.9±7.1	73.2±6.0	73.2±6.0	73.4±13.1	84.2±18.2	78.1±3.1	80.1±7.9	71.8±11.8	74.8±3.8	95.8±6.1	69.2±8.5	73.3±7.7
	6	61.9±6.6	74.7±6.6	74.7±6.6	65.8±6.4	73.8±9.1	94.6±8.2	76.7±7.9	78.8±10.1	75±4.8	84.3±7.7	72.3±9.9	80.0±10.8
**4**	7	-	-	-	-	69.0±12.4	91.5±1.0	62.6±7.6	47.6±0.0	71.7±5.8	111.4±53.5	85.5±8.5	78.4±5.9
	8	94.3±31.8	82.4±3.6	82.4±3.6	80.1±17.2	87.5±10.8	77.6±5.9	76.2±3.5	109.3±35.1	58.5±19.0	94.4±5.4	75.6±1.8	91.8±29.7
**5**	9	89.7±3.2	90.9±10.5	90.9±10.5	93.0±3.8	77.3±11.3	78.1±6.2	61.4±1.9	63.5±5.2	95.5±8.2	84.5±7.5	49.7±2.5	40.9±2.2
	10	77.9±6.5	92.8±4.1	92.8±4.1	106.6±12.6	69.9±11.0	81.4±6.7	98.9±4.7	85.4±6.9	89.1±9.2	81.5±7.1	56.2±12.5	92±13.8
**6**	11	60.6±7.7	79.3±7.8	79.3±7.8	65.2±12.1	-	-	-	-	56.8±9.6	96.0±11.9	64.9±13.1	80.2±4.1
	12	77.6±14.0	69.7±0.0	69.7±0.0	61.9±3.6	-	-	-	-	67.6±9.8	58.7±6.7	85.2±3.2	111.6±9
**7**	13	71.5±6.8	80.7±2.5	80.7±2.5	73.1±2.2	63.5±12.4	97.9±2.0	29.4±0.1	97.3±14.7	68.0±0.8	92.2±6.3	65.1±3.5	84.8±5.2
	14	90.2±11.3	62.3±8.1	62.3±8.1	87.4±0.2	81.6±11.7	80.1±4.4	71.9±6.8	67.9±12.5	75.6±3.1	-	66.2±23.7	81.9±2.9
**8**	15	39.6±7.9	61.1±7.7	61.1±7.7	64.2±1.1	122.9±6.7	113.1±0.7	113.0±1.0	86.2±11.5	63.0±11.9	79.1±6.5	54.5±3.6	67.7±1.0
	16	88.0±7.8	76.0±6.9	76.0±6.9	72.6±6.1	87.5±6.7	85.6±18.4	73.6±15.3	71.0±10.4	76.9±3.0	90.2±6.8	81.5±1.7	81.7±2.2
**9**	17	74.2±12.5	72.0±5.9	72.0±5.9	33.0±1.6	90.9±13.3	57.2±18.8	60.5±2.1	41.3±7.9	49.7±14.6	50.4±1.4	47.5±5.1	59.2±7.3
	18	79.5±2.5	72.6±4.1	72.6±4.1	77.2±9.1	65.0±3.3	53.9±10.7	76.7±0.1	52.3±7.4	67.6±7.7	69.5±2.4	65.3±10.6	72.8±8.2
**10**	19	81.9±9.6	79.9±5.5	79.9±5.5	84.2±4.6	60.7±21.6	88.5±9.0	75.5±4.5	67.1±11.2	81.0±10.1	97.6±4.6	71.7±0.9	73.1±1.9
	20	62.5±7.7	39.2±17.5	39.2±17.5	67.8±4.6	66.2±6.5	46.9±23.0	76.9±2.7	82.4±1.2	76±8.8	75.4±11.5	86.4±0.3	84.6±14.6
**M**		76.014	76.583	71.157	73.239	76.606	82.324	71.896	70.645	73.334	83.455	70.372	78.537
**SD**		17.121	14.923	15.567	15.320	17.964	17.694	19.307	17.220	13.614	14.946	12.160	15.677
**CV**		0.225	0.195	0.219	0.209	0.234	0.215	0.269	0.244	0.186	0.179	0.173	0.200
**p (r)**		0.863		0.627		0.219		0.820		**0.013 (0.55)**		**0.007 (0.57)**	

Displayed are the arithmetic means and standard deviations (M ± SD) of the amplitude variation (%) of the MMG and MTG signals of the triceps brachii muscle (MMGtri) and its tendon (MTGtri) as well as of the abdominal external oblique muscle (MMGobl) during the 15s and fatiguing trials comparing PIMA vs. HIMA. The group means, SD, coefficients of variation (CV), p-values and, in case of significance, the effect size r of statistical comparisons between HIMA and PIMA are given. Significant results are written in bold.

### Frequency and power

During the 15s and fatiguing trials, the arithmetic mean of mean frequency showed no significant differences in the paired t-test between PIMA and HIMA (*p* > 0.05, Table 1). The CV of frequency between the fatiguing trials was significantly higher during HIMA compared to PIMA, however, this only occurred for the MMGtri (*W* = 2.575, *p* = 0.010, *r* = 0.59) (M and SD of each participant can be found in the supplementary material).

**Fig 5:**
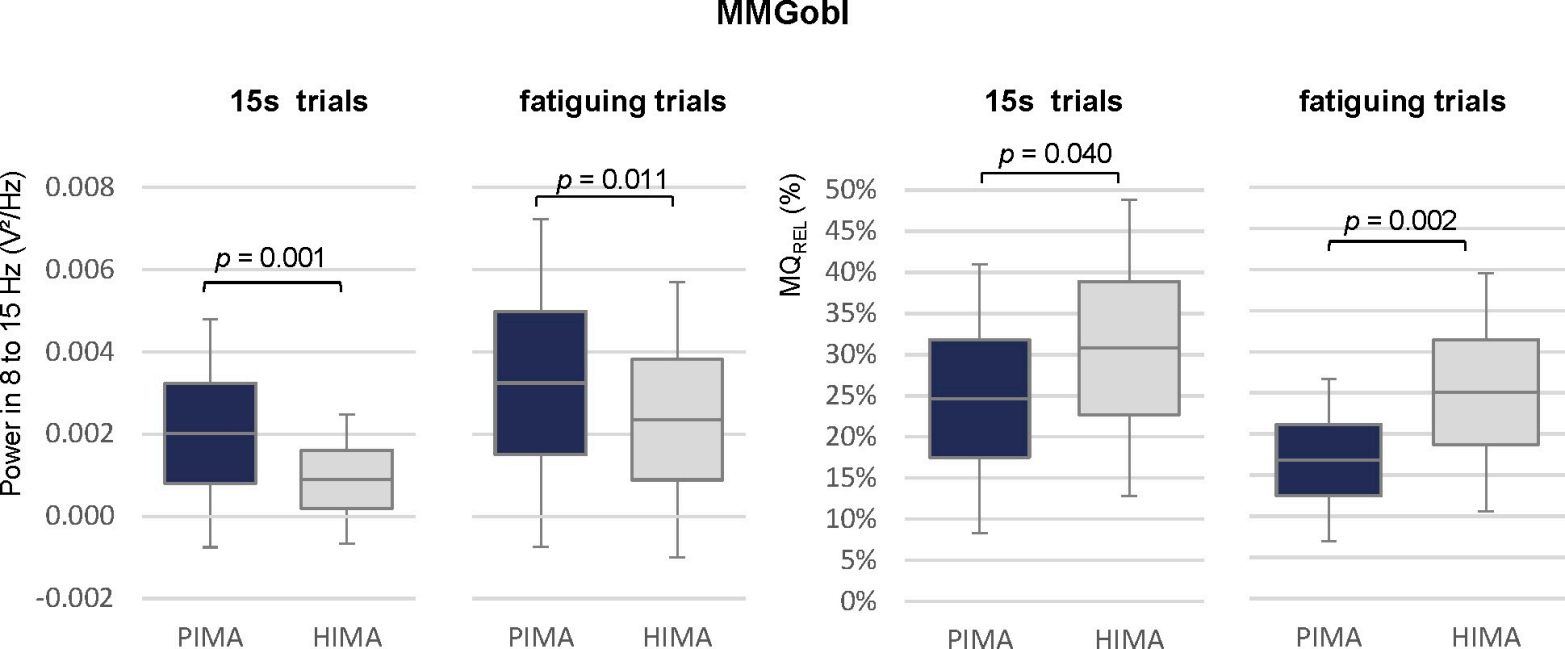
Power in 8 to 15 Hz and power frequency ratio MQ_REL_. Displayed are the arithmetic means, standard deviations (error bars) and the 95%-confidence intervals of the power in the frequency range of 8 to 15 Hz (left) and of the power frequency ratio MQ_REL_ of the MMGobl signal during the 15s and fatiguing trials for the tasks PIMA (blue) and HIMA (grey). Statistical comparisons between PIMA and HIMA revealed significant results for all comparisons with *p* = 0.001 to 0.04 and effect sizes of *r* = 0.47 to 0.76.

Considering the frequency range of 8 to 15 Hz the power differed significantly for MMGobl during the 15s-trials (*W* = -3.300, *p* = 0.001, *r* = 0.76) and during the fatiguing trials (*W* = -2.539, *p* = 0.011, *r* = 0.57), whereby PIMA showed higher values than HIMA (see Table 3, Fig 5). MMGtri and MTGtri showed no significances thereby (*p* > 0.05).

**Table 3:** Power in frequency ranges from 8 to 15 Hz.

		MMGtri				MTGtri				MMGobl			
		15s		fatiguing		15s		fatiguing		15s		fatiguing	
Couples		PIMA	HIMA	PIMA	HIMA	PIMA	HIMA	PIMA	HIMA	PIMA	HIMA	PIMA	HIMA
**1**	1	0.026±0.014	0.032±0.016	0.150±0.029	0.127±0.046	0.051±0.018	0.018±0.003	0.014±0.012	0.022±0.003	0.008±0.003	0.004±0.001	0.010±0.001	0.013±0.012
	2	0.035±0.008	0.016±0.006	0.042±0.006	0.059±0.032	0.022±0.008	0.005±0.002	0.074±0.006	0.028±0.006	0.000±0.000	0.000±0.000	0.003±0.001	0.003±0.000
**2**	3	0.001±0.001	0.000±0.000	0.004±0.000	0.006±0.006	0.000±0.000	0.000±0.000	0.004±0.004	0.002±0.002	0.000±0.000	0.000±0.000	0.001±0.001	0.000±0.000
	4	0.003±0.001	0.009±0.004	0.028±0.002	0.023±0.002	0.001±0.000	0.000±0.000	0.001±0.000	0.002±0.002	0.000±0.000	0.000±0.000	0.000±0.000	0.000±0.000
**3**	5	0.001±0.000	0.001±0.000	0.004±0.002	0.007±0.000	0.000±0.000	0.000±0.000	0.000±0.000	0.002±0.002	0.000±0.000	0.000±0.000	0.000±0.000	0.000±0.000
	6	0.003±0.002	0.000±0.000	0.015±0.011	0.006±0.002	0.000±0.000	0.000±0.000	0.002±0.000	0.000±0.000	0.000±0.000	0.000±0.000	0.001±0.000	0.000±0.000
**4**	7	-	-	-	-	0.029±0.012	0.010±0.005	0.012±0.008	0.024±	0.000±0.000	0.001±0.001	0.000±0.000	0.000±0.000
	8	0.007±0.006	0.006±0.002	0.011±0.003	0.002±0.003	0.074±0.082	0.059±0.041	0.052±0.071	0.014±0.007	0.005±0.005	0.002±0.001	0.002±0.002	0.001±0.001
**5**	9	0.001±0.000	0.002±0.000	0.003±0.001	0.004±0.001	0.003±0.001	0.002±0.001	0.005±0.001	0.011±0.000	0.000±0.000	0.000±0.000	0.000±0.000	0.001±0.000
	10	0.001±0.001	0.001±0.000	0.003±0.002	0.001±0.000	0.003±0.001	0.002±0.000	0.001±0.000	0.001±0.000	0.000±0.000	0.000±0.000	0.004±0.003	0.001±0.000
**6**	11	0.129±0.053	0.047±0.005	0.170±0.087	0.075±0.044	-	-	-	-	0.005±0.001	0.000±0.000	0.005±0.002	0.001±0.001
	12	0.060±0.0480	0.089±0.010	0.084±0.035	0.098±0.048	-	-	-	-	0.001±0.000	0.000±0.000	0.000±0.000	0.000±0.000
**7**	13	0.005±0.001	0.003±0.001	0.021±0.003	0.002±0.000	0.008±0.003	0.001±0.000	0.038±0.004	0.001±0.000	0.007±0.001	0.002±0.001	0.013±0.002	0.007±0.002
	14	0.005±0.002	0.003±0.001	0.009±0.002	0.012±0.002	0.007±0.001	0.008±0.001	0.017±0.014	0.023±0.011	0.001±0.000	-	0.004±0.002	0.003±0.000
**8**	15	0.010±0.005	0.003±0.001	0.016±0.008	0.006±0.001	0.000±0.000	0.000±0.000	0.001±0.000	0.002±0.001	0.001±0.000	0.000±0.000	0.002±0.000	0.001±0.000
	16	0.004±0.001	0.003±0.001	0.006±0.000	0.008±0.006	0.004±0.001	0.002±0.001	0.011±0.008	0.018±0.018	0.001±0.000	0.000±0.000	0.001±0.000	0.001±0.001
**9**	17	0.005±0.002	0.007±0.004	0.013±0.008	0.038±0.019	0.005±0.001	0.019±0.008	0.029±0.004	0.031±0.022	0.000±0.000	0.000±0.000	0.000±0.000	0.003±0.001
	18	0.031±0.008	0.016±0.004	0.036±0.045	0.016±0.000	0.052±0.050	0.023±0.007	0.008±0.002	0.008±0.004	0.003±0.001	0.001±0.000	0.005±0.002	0.002±0.001
**10**	19	0.038±0.003	0.052±0.006	0.051±0.006	0.051±0.005	0.011±0.003	0.004±0.001	0.006±0.000	0.003±0.001	0.000±0.000	0.000±0.000	0.001±0.000	0.001±0.000
	20	0.127±0.033	0.252±0.049	0.066±0.023	0.116±0.008	0.007±0.002	0.023±0.016	0.005±0.000	0.011±0.003	0.007±0.002	0.006±0.000	0.012±0.001	0.008±0.004
**M**		0.026	0.029	0.039	0.035	0.015	0.010	0.016	0.011	0.002	0.001	0.003	0.002
**SD**		0.040	0.059	0.049	0.041	0.022	0.015	0.020	0.011	0.003	0.002	0.004	0.003
**CV**		1.533	2.071	1.262	1.198	1.43	1.508	1.304	0.932	1.373	1.744	1.228	1.422
**p (r)**		0.717		0.601		0.053		0.679		**0.001 (0.76)**		**0.011 (0.57)**	

Displayed are the arithmetic means and standard deviations (M ± SD) of the power in the frequency range of 8 to 15 Hz (V^2^/Hz) of the MMG and MTG signals of the triceps brachii muscle (MMGtri) and its tendon (MTGtri) as well as of the abdominal external oblique muscle (MMGobl) during the 15s and fatiguing trials comparing PIMA vs. HIMA. The group means, SD, coefficients of variation (CV), p-values and, in case of significance, the effect size r of statistical comparisons between HIMA and PIMA are given. Significant results are written in bold.

Furthermore, the power frequency ratio MQ_REL_ showed significantly higher values in the MMGobl during HIMA compared to PIMA during the fatiguing trials (t(19) = -3.673, *p* = 0.002, *r* = 0.64) and during the 15s-trials (*W* = 2.052, *p* = 0.040, *r* = 0.47) (Table 4, Fig 5). Thereby, during HIMA, the lower frequency range of 3 to 7 Hz amounted averagely 24.9 ± 14.6% of the whole range of 3 to 12 Hz during the fatiguing trials and averagely 30.8 ± 18.0% during 15s-trials. Whereas during PIMA, the lower frequency range amounted averagely 16.7 ± 9.9% of the whole frequency range of 3 to 12 Hz during the fatiguing trials and 24.7 ± 16.3% during the 15s-trials. The signals of MMGtri and MTGtri did not differ significantly concerning those parameters (*p* > 0.05) (Table 4).

**Table 4:** Power-frequency ratio MQ_REL_.

		MMGtri				MTGtri				MMGobl			
		15s		fatiguing		15s		fatiguing		15s		fatiguing	
Couples		PIMA	HIMA	PIMA	HIMA	PIMA	HIMA	PIMA	HIMA	PIMA	HIMA	PIMA	HIMA
**1**	1	0.09 ± 0.03	0.18 ± 0.03	0.10 ± 0.03	0.15 ± 0.00	0.11 ± 0.04	0.13 ± 0.02	0.09 ± 0.02	0.07±0.01	0.07 ± 0.05	0.15 ± 0.03	0.13 ± 0.01	0.16 ± 0.12
	2	0.09 ± 0.01	0.24 ± 0.09	0.13 ± 0.01	0.08 ± 0.01	0.08 ± 0.01	0.27 ± 0.04	0.07 ± 0.03	0.1±0.03	0.10 ± 0.02	0.18 ± 0.04	0.03 ± 0.00	0.04 ± 0.02
**2**	3	0.62 ± 0.08	0.73 ± 0.07	0.38 ± 0.13	0.43 ± 0.02	0.52 ± 0.11	0.58 ± 0.10	0.22 ± 0.06	0.26±0.05	0.37 ± 0.1	0.51 ± 0.09	0.28 ± 0.12	0.58 ± 0.07
	4	0.32 ± 0.02	0.43 ± 0.11	0.32 ± 0.07	0.35 ± 0.06	0.46 ± 0.01	0.48 ± 0.12	0.34 ± 0.02	0.23±0.08	0.31 ± 0.05	0.34 ± 0.03	0.31 ± 0.05	0.30 ± 0.05
**3**	5	0.23 ± 0.19	0.22 ± 0.08	0.28 ± 0.03	0.25 ± 0.11	0.42 ± 0.12	0.29 ± 0.05	0.25 ± 0.04	0.15±0.03	0.24 ± 0.12	0.39 ± 0.07	0.18 ± 0.01	0.12 ± 0.04
	6	0.19 ± 0.04	0.25 ± 0.03	0.16 ± 0.04	0.12 ± 0.01	0.29 ± 0.08	0.47 ± 0.14	0.25 ± 0.00	0.27±0.04	0.21 ± 0.03	0.19 ± 0.04	0.11 ± 0.02	0.24 ± 0.04
**4**	7	-	-	-	-	0.12 ± 0.09	0.32 ± 0.15	0.13 ± 0.05	0.05±	0.80 ± 0.02	0.81 ± 0.04	0.12 ± 0.01	0.19 ± 0.13
	8	0.55 ± 0.21	0.39 ± 0.05	0.35 ± 0.05	0.42 ± 0.17	0.54 ± 0.13	0.50 ± 0.04	0.56 ± 0.31	0.49±0.20	0.24 ± 0.07	0.43 ± 0.10	0.36 ± 0.05	0.45 ± 0.12
**5**	9	0.29 ± 0.03	0.30 ± 0.02	0.31 ± 0.00	0.36 ± 0.07	0.25 ± 0.08	0.31 ± 0.07	0.32 ± 0.10	0.28±0.08	0.44 ± 0.05	0.27 ± 0.02	0.10 ± 0.01	0.08 ± 0.03
	10	0.25 ± 0.04	0.38 ± 0.04	0.16 ± 0.02	0.51 ± 0.10	0.33 ± 0.06	0.41 ± 0.02s	0.49 ± 0.22	0.63±0.07	0.27 ± 0.07	0.38 ± 0.07	0.11 ± 0.05	0.36 ± 0.01
**6**	11	0.12 ± 0.08	0.16 ± 0.05	0.06 ± 0.03	0.14 ± 0.12	-	-	-	-	0.14 ± 0.05	0.49 ± 0.04	0.16 ± 0.09	0.31 ± 0.16
	12	0.33 ± 0.15	0.18 ± 0.01	0.29 ± 0.12	0.14 ± 0.01	-	-	-	-	0.28 ± 0.08	0.12 ± 0.01	0.37 ± 0.02	0.45 ± 0.04
**7**	13	0.23 ± 0.03	0.31 ± 0.04	0.14 ± 0.01	0.3 ± 0.00	0.12 ± 0.06	0.38 ± 0.08	0.02 ± 0.00	0.42±0.03	0.24 ± 0.02	0.39 ± 0.04	0.16 ± 0.03	0.35 ± 0.05
	14	0.46 ± 0.05	0.32 ± 0.06	0.46 ± 0.01	0.43 ± 0.01	0.33 ± 0.11	0.20 ± 0.03	0.3 ± 0.03	0.26±0.01	0.28 ± 0.07	-	0.17 ± 0.15	0.40 ± 0.01
**8**	15	0.07 ± 0.04	0.15 ± 0.09	0.05 ± 0.04	0.16 ± 0.04	0.69 ± 0.05	0.61 ± 0.03	0.59 ± 0.02	0.67±0.07	0.17 ± 0.04	0.29 ± 0.07	0.06 ± 0.02	0.17 ± 0.03
	16	0.26 ± 0.05	0.19 ± 0.02	0.18 ± 0.09	0.10 ± 0.03	0.33 ± 0.05	0.43 ± 0.02	0.40 ± 0.09	0.26±0.04	0.15 ± 0.03	0.28 ± 0.01	0.17 ± 0.06	0.17 ± 0.00
**9**	17	0.20 ± 0.08	0.25 ± 0.01	0.23 ± 0.01	0.08 ± 0.03	0.42 ± 0.16	0.21 ± 0.11	0.20 ± 0.11	0.09±0.01	0.04 ± 0.06	0.02 ± 0.01	0.01 ± 0.00	0.03 ± 0.03
	18	0.20 ± 0.02	0.22 ± 0.05	0.20 ± 0.07	0.25 ± 0.04	0.13 ± 0.03	0.12 ± 0.04	0.35 ± 0.02	0.16±0.04	0.18 ± 0.04	0.12 ± 0.01	0.16 ± 0.03	0.21 ± 0.07
**10**	19	0.28 ± 0.03	0.22 ± 0.04	0.18 ± 0.04	0.25 ± 0.04	0.18 ± 0.13	0.38 ± 0.11	0.24 ± 0.02	0.24±0.03	0.25 ± 0.08	0.30 ± 0.03	0.14 ± 0.04	0.17 ± 0.01
	20	0.19 ± 0.03	0.16 ± 0,07	0.27 ± 0.07	0.20 ± 0.03	0.22 ± 0.07	0.16 ± 0.13	0.26 ± 0.01	0.18±0.08	0.14 ± 0.02	0.19 ± 0.06	0.23 ± 0.02	0.21 ± 0.06
**M**		0.262	0.278	0.225	0.248	0.307	0.348	0.283	0.267	0.247	0.308	0.167	0.249
**SD**		0.149	0.137	0.111	0.134	0.175	0.149	0.158	0.179	0.163	0.180	0.099	0.146
**CV**		0.569	0.492	0.494	0.540	0.569	0.429	0.560	0.670	0.662	0.585	0.592	0.587
**p (r)**		0.421		0.370		0.208		0.199		**0.040 (0.47)**		**0.002 (0.64) **	

Displayed are the arithmetic means and standard deviations (M ± SD) of the power frequency ratio MQ_REL_ of the MMG and MTG signals of the triceps brachii muscle (MMGtri) and its tendon (MTGtri) as well as of the abdominal external oblique muscle (MMGobl) during the 15s and fatiguing trials comparing PIMA vs. HIMA. The group means, SD, coefficients of variation (CV), p-values and, in case of significance, the effect size r of statistical comparisons between HIMA and PIMA are given. Significant results are written in bold.

## Discussion

In the present study, two tasks of isometric muscle action were performed in a paired couple setting, whereby each of the two partners performed either the pushing isometric motor task (PIMA) or the holding isometric motor task (HIMA). The tasks changed between the trials, so that each partner performed each task in the same couple. The main findings were: (1) The subjects who performed PIMA were able to maintain the force level for longer, the categorization “HIMA failure” arose in 85% of the 40 fatiguing trials. This supports the previous findings concerning the briefer time to task failure during HIMA vs. PIMA and the distinction of two forms of isometric muscle action regarding the submaximal isometric endurance. (2) The mean frequency and normalized mean amplitude showed no significant differences between PIMA and HIMA for all signals in 15s and fatiguing trials as was expected on the base of previous investigations. (3) MMGtri and MTGtri showed no significant differences between PIMA and HIMA regarding the other oscillatory parameters (amplitude variation, power and MQ_REL_). The MMGobl showed (4) a significantly higher amplitude variation during HIMA, (5) a significantly higher power in the frequency range of 8 to 15 Hz during PIMA and (6) a significantly higher power frequency ratio (MQ_REL_) during HIMA compared to PIMA for the 15s and fatiguing trials. This speaks for different patterns of the mechanical muscle oscillations of the abdominal external oblique muscle comparing HIMA and PIMA in the interpersonal setting. However, the MMG/MTG signals of the triceps brachii muscle and its tendon, respectively, do not reflect these patterns. Possible explanations for those results are discussed after presenting the limitations.

### Methodological limitations

The present setting of muscular interaction, whereby one partner performed HIMA and the other one performed PIMA, is a novel approach. The inspection of the performed motor tasks is difficult. This limitation was tried to be controlled by the design that only the pushing partner had the visual control of the force level and, therefore, should act and initiate the force isometrically in direction of elbow extension. The holding partner should just react isometrically and, thereby, only had a kinesthetic control. Therefore, the task instructions were given in a pronounced way to ensure the correct performances of both tasks. However, it could not be ruled out completely that a subject switched unconsciously between the tasks HIMA and PIMA in the course of measurement. As mentioned above, one participant contacted the border during HIMA. This was not expected since, thereby, the subject extended its elbow joint although this is not intended during HIMA. That is why we assume that this participant switched into PIMA in the course of the interaction. The other partner yielded and, thus, flexed his elbow. Thus, an eccentric muscle action must have been performed. This should not occur during PIMA–per definitionem. For what reason this outcome appeared, can only be discussed hypothetically. The MVIC was nearly identical between both partners (partner A: 33.18 Nm vs. B: 32.90 Nm). Since this was the only exception, we assume that the tasks were performed largely adequate due to the following reasons: the tasks instructions, the setting (control of force level only by the pushing subject, the holding one just reacted to the partners force application) and the fact that the border was contacted in most cases by the pushing partner–irrelevant of the physical condition of the partners.

The results might have been influenced due to the visual feedback for the partner who performed PIMA. There are inconclusive opinions and findings concerning the influence of a visual feedback on force variability or EMG activity [14,15]. Since HIMA and PIMA did not differ regarding the normalized amplitude of MMG/MTG signals this would speak against an influence of the feedback on the results. Furthermore, the previously performed investigation with single measurements [1] showed significant differences between HIMA and PIMA although the visual feedback was identical for both tasks. However, a possible influence of the visual feedback on the results cannot be completely ruled out.

Another limitation of the present study was that during the interaction, the partners sometimes slipped, so that the measurement had to be repeated. This was due to the connection with the strain gauge, the oscillating character of the interaction and the high force level. For further investigations, a reduction of force intensity might help solving this problem. Since the slipping parts were excluded from evaluation, this limitation should not have influenced the results.

Furthermore, due to the different physical conditions, the force level was not always similar for both partners. This is a limitation, which is difficult to solve in a paired setting. However, since the tasks changed, this limitation was controlled. Nevertheless, the results that the pushing partner mostly could maintain the task–irrespective of the physical condition–is even more impressive due to the different physical conditions. For further investigations, the MVIC could be measured during interaction between the partners, whereby both partners perform PIMA, so that the maximal reaction force of the pair would be regarded as MVIC. There are pros and cons for both alternatives.

Four MMG/MTG-signals had to be excluded, since the sensors failed. This could have been controlled better in the course of the measurements. However, due to the high number of MMG- and MTG-sensors, there was no opportunity to exchange the piezoelectric sensors during the measuring.

A multiple test problem could arise for critical statisticians. Since the study has an explorative character, this limitation is discussible. Many researchers take the view that multiple testing is permitted in this case [16–19]. However, the results have to be interpreted with caution–also because of the small sample size of 10 pairs.

### Capability of maintaining the tasks PIMA or HIMA

It is noteworthy that the results were obtained in a setting, in which the tasks changed in the same couple. Thus, depending on the tasks HIMA or PIMA, the capacity of maintaining the identical force level changed–regardless of which partner was physically stronger and showed a higher MVIC. This was also apparent for couples with very high differences in MVIC, e.g., in one couple the stronger partner had a MVIC of 159% of the MVIC of the weaker. That means he performed the fatiguing trials with 53% of his MVIC. Nevertheless, he failed during the HIMA trials. The result that 34 “HIMA failures” occurred in a total amount of 40 trials speaks for a better capability of maintaining the task during PIMA. This suggests a better submaximal isometric endurance during a pushing isometric action compared to a holding one with identical force intensities. Because of the randomized order of the allocation of the tasks, an effect of the physical conditions can be ruled out as reason for the results of longer maintaining time in PIMA. Only five “PIMA failures” occurred, whereby just one subject contacted the border in two trials (see limitations).

There are different possible explanations for the decreased capability of maintaining the HIMA task, e.g., a metabolic fatigue, effects of ischemia or higher demands of neural control. Basically, a metabolic fatigue seems to be less obvious since the intensity and muscle length during the isometric muscle action were similar for both tasks. Nevertheless, a possible higher demand of energetic substances or a higher production of lactate during HIMA would be conceivable. In a study of Rudroff et al. [6] a higher glucose uptake (PET method) of the lower extremity muscles was found in 11 of 24 muscles after fatiguing trials at 25% of MVIC performing HIMA compared to PIMA in young male (*n* = 3), but not for older male participants (*n* = 3). This would speak for a higher consumption of glucose during HIMA in young men and might reduce the submaximal force endurance capability during HIMA. Another explanation for the shorter endurance time during HIMA could be the capillary behavior. There seems to be different types of capillary behavior with regard to blood filling and oxygen saturation as shown in Dech et al. [20] during holding a weight for as long as possible. This might change for PIMA. However, in a study of Booghs et al. [21] no difference in muscle oxygenation of the elbow flexors at 20 and 60% of the MVIC occurred comparing HIMA vs. PIMA. Since the muscle oxygenation was not considered in the presented study, no statement can be made concerning this parameter. A central fatigue due to higher neuronal demands might be a conceivable explanation for the briefer endurance time during HIMA.

### Possible higher demands of neuronal control during HIMA compared to PIMA

It was supposed that HIMA requires higher neural control strategies compared to PIMA [1], since during HIMA the endurance time was significantly shorter compared to PIMA in several investigations and in the present study [1,4–7]. In the present study, the myofascial oscillations of the investigated muscles showed no differences between HIMA and PIMA concerning the mean frequency and mean amplitude of all signals. This was hypothesized on the base of the former MMG/MTG study during single measurements [1] as well as on other EMG studies [2–5] investigating HIMA and PIMA. The commonly considered parameters of frequency and amplitude of EMG or MMG/MTG, respectively, seem not to provide a suitable approach to distinguish HIMA and PIMA. This underlines the necessity to consider other parameters, provided that there is a difference in performing those tasks, which is reflected by the submaximal force endurance.

Assuming different neural control strategies, it might be helpful to look into parameters, which reflect more complex characterizations, as e.g., the amplitude variation or the relation of the power in different frequency bands (MQ_REL_). Since these parameters were newly developed in investigations with Parkinson patients without tremor [8], those parameters were not evaluated before in this context. In the present study, the oscillatory parameters as the amplitude variation and the power-frequency ratio MQ_REL_ showed differences for the MMGobl (not for MMGtri/MTGtri). Theoretical considerations to explain these results might be found in neuroscientific research. Therein, it is assumed that the more adaptation is required during a motor task, the more relevant the feedback control gets [22,23]. During HIMA, the participant has to react to the force of the partner, which performs PIMA. The pushing partner just has to apply the force level onto the counterpart and has not to react kinesthetically as intense as during HIMA. Therefore, it might be conceivable that HIMA entails higher requirements regarding the adaptive processes based on kinesthetic perception compared to PIMA. Hence, the thereby assumed more difficult neural control might explain the briefer endurance time during the holding task.

External variations, as a varying external force impact, require a constant update of the sensorimotor system [24]. To execute motor tasks appropriately an adequate adaptability is necessary. With a more complex feedback control, the variability will be even higher as a sign of an adequate adaptation [25]. In the present study, the amplitude variation of the mechanical muscular oscillations of the abdominal external oblique muscle were significantly higher during HIMA with 83 ± 15% variation during the 15s trials and 79 ± 16% during the fatiguing trials compared to PIMA with 73 ± 14% and 70 ± 12%, respectively (*p* = 0.013, *r* = 0.55; *p* = 0.007, *r* = 0.57). This might be a sign for higher adaptation processes and, therefore, for a more complex control strategy during HIMA.

Furthermore, the power frequency ratio of the power in the low frequency range of 3 to 7 Hz related to the power in the area of 3 to 12 Hz might reflect more complex control strategies if the lower frequencies are represented more pronounced. In the present study, the power in the frequency range of 3 to 7 Hz amounted to 25 ± 15% during HIMA in the fatiguing trials and 17 ± 10% during PIMA (*p* = 0.002, *r* = 0.64) for the MMGobl. This might point out that the power distribution is changed during HIMA in a broader range, which possibly could enable the subject to react more appropriate to external changes as they were applied by the partner, who initiated and controlled the force level. These considerations are still speculative but seem to be conceivable and not that unlikely based on the reasonable neuroscientific explanations.

In looking more precisely at the performance of tasks HIMA and PIMA, we assume that HIMA is closer to eccentric muscle action and PIMA reflects rather the concentric muscle action–both in the sense of a “stopped” eccentric or concentric muscle action, respectively. This was hypothesized by Garner et al. [2] However, they rejected the hypothesis since they found no significant differences between HIMA und PIMA concerning the EMG amplitude and frequency. As mentioned above, probably other parameters of the muscular output, as e.g., the amplitude variation or the power frequency ratio, respectively, could be more suitable to reflect the neuronal control processes more precisely. During HIMA, the participant just should react to the partner, but in case of exceeding the maximal holding endurance under submaximal intensities, this partner would yield in direction of eccentric muscle action, thus, the muscle would lengthen. During PIMA, however, the participant works in direction of concentric muscle action. In case of a declining resistance offered by the partner in the course of time, the pushing subject would shorten its muscle in order to maintain the reaction force level, thus, would perform concentric muscle action. This leads to the assumption that HIMA might reflect the control processes of eccentric and PIMA those of concentric muscle action. This could further underpin the hypothesis of more complex control strategies during HIMA, since several investigations suggest a higher complexity of the neural control during eccentric muscle actions [26–29]. This is not at least based on the findings, that during eccentric muscle action the cortical potential is higher compared to the concentric contraction [30]. However, the muscular activity measured by EMG is higher during concentric muscle action compared to eccentric one [28,30–37]. The results that the normalized amplitude tends to be higher and the power in the frequency range of 8 to 15 Hz is significantly higher for the MMGobl (not for MMGtri and MTGtri) during PIMA in the fatiguing trials might, therefore, support the assumption that PIMA is closer to concentric muscle action. In contrast, during HIMA the power in the same frequency range is lower, which might reflect the lower muscular activity during an eccentric muscle action. If a higher supraspinal activity is in fact apparent during HIMA remains open. This question has to be investigated by using methods, which are able to capture the supraspinal processes. Measurements using EEG, EMG and MMG were performed in our Neuromechanics Lab, but the evaluation is still pending.

### Considerations concerning the different findings between MMGtri, MTGtri and MMGobl

It has to be questioned, why the signals of the triceps brachii and its tendon did not show any significant differences, although this muscle group had to execute the motor tasks with respect to the elbow joint. The most conceivable reason might be that the forearms of both partners were coupled. Thereby, a synchronized mutual rhythm arises between both partners [9,10]. A coupling like this can occur basically in different ways [13]: Firstly, a master slave relation might arise, whereby one partner is active and the other one is passive. Since both neuromuscular systems are active, this coupling manner can be ruled out here. Secondly, both partners could agree on a mutual rhythm, whereby a distinctive interaction frequency would be generated. Thirdly, a leader-follower relation might arise, whereby one partner follows the other one. Since in the present setting the pushing partner initiates the force and the holding partner should react to this, we assume that a leader-follower constellation arises. For further discussion and mathematical reasoning see Schaefer & Bittmann [9,10]. Thereby, the muscular oscillations might have been transferred through the interface onto the other partner and might have diminished possible differences in the oscillatory behavior of MMG and MTG of the triceps brachii muscle and its tendon. Since one partner performed HIMA while the other one performed PIMA, the specific characteristics of each tasks might have been eliminated thereby. A technical crosstalk of the sensors can be excluded because of the characteristics of piezo-electric sensors (high-impedance, minimal current, shielded) and the distance between the sensors in the setting. The external abdominal oblique muscle was the most distal recorded muscle from the coupling point and, therefore, was not directly coupled between the partners. That might be a possible explanation why only the MMGobl showed significant differences between PIMA and HIMA. This might be further supported by a study in the same setting, whereby the oscillations of the MMGtri, MTGtri and MMGobl signals showed interpersonal coherent behavior [10]. The coherence was highest for MTGtri and MMGtri. MMGobl showed also significant coherence, however, the duration of coherent phases was briefer.

In the previous study concerning two forms of isometric muscle action during single measurements [1], the power in the frequency range of 8 to 15 Hz and 10 to 29 Hz was higher during HIMA compared to PIMA for the MTG signal of triceps tendon. This indicates that also the executing muscle-tendon structures show differences with regard to HIMA and PIMA if a coupling is not present. However, in the study of Rudroff et al. [5] investigating both motor tasks during muscular action of the elbow flexors at 20, 30, 45 and 60% of the MVIC using EMG, the power in the range of 10 to 29 Hz towards the end of measurement behaved reversed with higher amounts in PIMA compared to HIMA. This is consistent with the here presented results that the power in the frequency range of 8 to 15 Hz was higher during PIMA vs. HIMA for the MMGobl. The contrary findings of the previous study investigating PIMA and HIMA in a setting with single measurements [1] might be due to the setting or to the regarded structure. It is conceivable that the tendon’s oscillations might behave differently since three muscle heads insert into the triceps tendon. This could result in a deviating oscillatory behavior. Further investigations have to examine this assumption.

The significant differences of amplitude variation, power in the frequency of 8 to 15 Hz and MQ_REL_ for the MMGobl between HIMA and PIMA might also be due to the role of the abdominal external oblique muscle as core stabilizer in the present setting. That indicates that the muscular core stabilization differs with respect to the motor tasks. In the former investigation with single measurements, this effect was not visible for the MMGobl [1]. However, thereby only the power in the frequency area of 8 to 15 Hz was considered, not the amplitude variation or the MQ_REL_. Probably, the interaction of two subjects enhances the effect of PIMA or HIMA on the oscillatory behavior of the abdominal external oblique muscle. However, the cause of the observed difference between HIMA and PIMA remains unclear.

## Conclusion

In the present setting, two participants interacted isometrically, whereby one partner performed the holding isometric task and the other one performed the pushing isometric task. The results showed that thereby differences between HIMA and PIMA occurred independent of the physical conditions of the subjects. Especially the endurance capability seems to be briefer, the amplitude variation and the specific power frequency ratio revealed higher and the power in the frequency range of 8 to 15 Hz lower values in the MMG of external abdominal oblique muscle during HIMA compared to PIMA. The MMG and MTG of the triceps brachii muscle and tendon, which are closer to the coupling interface between the partners, did not show this behavior. Future investigations have to examine whether or not the results can be supported, especially concerning the newly applied analysis of amplitude variation and the specific power frequency ratio.

Also, with regard to other investigations on this topic so far, the results support the hypothesis that two different forms of isometric muscle action exist. We suggest that the holding form requires higher neuronal control strategies, since a more adaptive functionality is demanded thereby. Probably, those higher requirements for adaptive processes could be an explanation of the observed reduced submaximal isometric endurance in HIMA. This is close to the considerations and the concept of the Adaptive Force [38,39]. Thereby, the neuromuscular system has to adapt isometrically to an increasing external force. If the maximal holding force is reached, the subject merges into an adapting eccentric muscle action. There are hints that the Adaptive Force is vulnerable for disturbances of the neural system, as e.g., mental or olfactory inputs (publication in work). This might have practical implications, especially in medicine but also concerning injury mechanisms.

It is assumed that HIMA is closer to eccentric muscle actions and PIMA is closer to concentric contraction including the underlying neuromuscular control strategies. We dare to hypothesize that isometric muscle action per se does not exist because oscillations are always present. This indicates an alternating slight shortening and lengthening of the muscle. Presumably, the intention to hold isometrically generates a different control strategy than the intention to push isometrically. We supposed to interpret PIMA as a “stopped” concentric and HIMA as a “stopped” eccentric muscle action, respectively. This would be a background rationale for the suggested higher demands of neuromuscular control during HIMA. Further basic research is needed to get more insights into the underlying mechanisms. In exercise and training the holding function is already getting more visibility [40]. The holding function might adopt a special status in sports and movement sciences, but also in neurosciences and medicine. As a first step, the two forms of isometric muscle action and their closeness to eccentric or concentric muscle action, respectively, should be investigated by additional research. In case of scientific confirmation, a gap in motor science should be filled since this topic is not considered therein until now.

## Supporting information

S1 Table Values of normalized amplitude. (PDF)

S2 Table Values of mean frequency. (PDF)
