# Supplementary material for: Paired personal interaction reveals objective differences between pushing and holding isometric muscle action
Source: PLoS One. 2021 May 6;16(5):e0238331. doi: 10.1371/journal.pone.0238331 (PMC8101915; doi:10.1371/journal.pone.0238331)
Supplement: S1 Table — (PDF) [file pone.0238331.s001.pdf]

Supplementary information

Schaefer & Bittmann

Title: Paired personal interaction reveals objective differences between pushing and holding isometric muscle action

S1 Table. Values of normalized amplitude.

S2 Table. Values of mean frequency.

S1 Table. **Values of normalized amplitude.** Arithmetic means (M) (± standard deviation (SD)) of the normalized amplitude of the mechanomyographic and mechanotendographic signals of the triceps brachii muscle (MMGtri) and its tendon (MTGtri) as well as of the abdominal external oblique muscle (MMGobl) during the 15s and fatiguing trials comparing PIMA vs. HIMA. The group M, SD, coefficient of variation (CV) and p-values of statistical comparisons between HIMA and PIMA are displayed. In case of significance, the effect size r is given.

		Normalized amplitude [%]											
		MMGtri				MTGtri				MMGobl			
		15s-trial		fatigue		15s-trial		fatigue		15s-trial		fatigue	
Couples		**PIMA**	**HIMA**	**PIMA**	**HIMA**	**PIMA**	**HIMA**	**PIMA**	**HIMA**	**PIMA**	**HIMA**	**PIMA**	**HIMA**
1	*1*	0.40 ± 0.08	0.28 ± 0.05	0.57 ± 0.00	0.47 ± 0.06	0.42 ± 0.03	0.29 ± 0.02	0.39 ± 0.05	0.38 ± 0.05	0.29 ± 0.02	0.25 ± 0.09	0.37 ± 0.07	0.35 ± 0.01
	2	0.38 ± 0.02	0.29 ± 0.04	0.41 ± 0.00	0.36 ± 0.03	0.36 ± 0.07	0.39 ± 0.03	0.42 ± 0.00	0.33 ± 0.01	0.28 ± 0.04	0.23 ± 0.07	0.31 ± 0.04	0.38 ± 0.06
2	*3*	0.22 ± 0.02	0.26 ± 0.06	0.31 ± 0.01	0.30 ± 0.00	0.32 ± 0.05	0.27 ± 0.04	0.36 ± 0.05	0.27 ± 0.02	0.28 ± 0.04	0.27 ± 0.05	0.24 ± 0.03	0.12 ± 0.01
	4	0.30 ± 0.02	0.31 ± 0.04	0.29 ± 0.03	0.32 ± 0.01	0.31 ± 0.02	0.25 ± 0.01	0.22 ± 0.05	0.29 ± 0.01	0.27 ± 0.03	0.26 ± 0.06	0.27 ± 0.06	0.21 ± 0.00
3	*5*	0.30 ± 0.05	0.34 ± 0.03	0.29 ± 0.04	0.27 ± 0.01	0.29 ± 0.09	0.28 ± 0.06	0.20 ± 0.04	0.21 ± 0.06	0.27 ± 0.05	0.16 ± 0.07	0.26 ± 0.05	0.27 ± 0.06
	6	0.31 ± 0.04	0.30 ± 0.03	0.29 ± 0.03	0.32 ± 0.01	0.19 ± 0.03	0.18 ± 0.08	0.20 ± 0.00	0.29 ± 0.03	0.33 ± 0.05	0.28 ± 0.05	0.21 ± 0.06	0.22 ± 0.09
4	*7*	-	-	-	-	0.29 ± 0.02	0.27 ± 0.04	0.30 ± 0.04	0.37 ±	0.32 ± 0.06	0.27 ± 0.05	0.28 ± 0.01	0.27 ± 0.01
	8	0.15 ± 0.07	0.26 ± 0.01	0.34 ± 0.10	0.32 ± 0.04	0.29 ± 0.17	0.32 ± 0.07	0.30 ± 0.08	0.22 ± 0.11	0.29 ± 0.06	0.21 ± 0.04	0.32 ± 0.07	0.30 ± 0.01
5	*9*	0.24 ± 0.03	0.29 ± 0.03	0.28 ± 0.02	0.27 ± 0.04	0.31 ± 0.02	0.28 ± 0.01	0.30 ± 0.03	0.45 ± 0.05	0.24 ± 0.03	0.29 ± 0.04	0.34 ± 0.06	0.45 ± 0.03
	10	0.30 ± 0.05	0.24 ± 0.03	0.42 ± 0.11	0.22 ± 0.05	0.30 ± 0.02	0.27 ± 0.01	0.30 ± 0.02	0.28 ± 0.04	0.23 ± 0.10	0.25 ± 0.03	0.38 ± 0.07	0.23 ± 0.05
6	*11*	0.46 ± 0.02	0.32 ± 0.04	0.56 ± 0.13	0.37 ± 0.10	-	-	-	-	0.33 ± 0.03	0.29 ± 0.05	0.35 ± 0.02	0.29 ± 0.03
	12	0.32 ± 0.17	0.43 ± 0.03	0.47 ± 0.02	0.48 ± 0.05	-	-	-	-	0.28 ± 0.08	0.34 ± 0.06	0.30 ± 0.07	0.27 ± 0.01
7	*13*	0.31 ± 0.04	0.31 ± 0.03	0.29 ± 0.14	0.28 ± 0.06	0.22 ± 0.02	0.26 ± 0.03	0.39 ± 0.08	0.23 ± 0.02	0.33 ± 0.02	0.31 ± 0.05	0.32 ± 0.02	0.26 ± 0.03
	14	0.23 ± 0.06	0.33 ± 0.04	0.17 ± 0.02	0.28 ± 0.02	0.26 ± 0.03	0.25 ± 0.03	0.26 ± 0.08	0.34 ± 0.05	0.30 ± 0.03	-	0.26 ± 0.01	0.16 ± 0.01
8	*15*	0.43 ± 0.03	0.33 ± 0.01	0.45 ± 0.09	0.30 ± 0.01	0.23 ± 0.03	0.20 ± 0.01	0.23 ± 0.04	0.24 ± 0.02	0.41 ± 0.04	0.32 ± 0.05	0.40 ± 0.03	0.22 ± 0.02
	16	0.30 ± 0.01	0.30 ± 0.04	0.27 ± 0.03	0.28 ± 0.05	0.24 ± 0.09	0.26 ± 0.05	0.24 ± 0.07	0.34 ± 0.03	0.26 ± 0.04	0.26 ± 0.02	0.29 ± 0.01	0.24 ± 0.10
9	*17*	0.34 ± 0.03	0.31 ± 0.05	0.23 ± 0.04	0.29 ± 0.05	0.27 ± 0.04	0.32 ± 0.02	0.30 ± 0.01	0.32 ± 0.06	0.29 ± 0.03	0.33 ± 0.07	0.29 ± 0.03	0.31 ± 0.04
	18	0.32 ± 0.04	0.33 ± 0.01	0.29 ± 0.08	0.30 ± 0.00	0.35 ± 0.02	0.36 ± 0.05	0.25 ± 0.04	0.33 ± 0.00	0.30 ± 0.05	0.35 ± 0.01	0.25 ± 0.01	0.27 ± 0.01
10	*19*	0.34 ± 0.01	0.37 ± 0.02	0.37 ± 0.01	0.36 ± 0.03	0.34 ± 0.06	0.31 ± 0.02	0.33 ± 0.00	0.32 ± 0.03	0.30 ± 0.05	0.28 ± 0.04	0.28 ± 0.01	0.29 ± 0.05
	20	0.50 ± 0.08	0.65 ± 0.09	0.40 ± 0.04	0.48 ± 0.05	0.34 ± 0.04	0.40 ± 0.11	0.24 ± 0.04	0.31 ± 0.06	0.34 ± 0.04	0.31 ± 0.03	0.23 ± 0.00	0.23 ± 0.02
	M	0.323	0.330	0.352	0.330	0.296	0.287	0.291	0.307	0.297	0.276	0.298	0.266
	SD	0.083	0.089	0.107	0.075	0.056	0.057	0.067	0.061	0.039	0.047	0.050	0.074
	CV	0.258	0.269	0.303	0.229	0.190	0.199	0.229	0.198	0.132	0.168	0.169	0.278
	p (r)	0.573		0.355		0.408		0.422		0.074		0.055 (0.43)	
