# Supplementary material for: Paired personal interaction reveals objective differences between pushing and holding isometric muscle action
Source: PLoS One. 2021 May 6;16(5):e0238331. doi: 10.1371/journal.pone.0238331 (PMC8101915; doi:10.1371/journal.pone.0238331)
Supplement: S2 Table — (PDF) [file pone.0238331.s002.pdf]

S2 Table. **Values of mean frequency.** Arithmetic means (M) (± standard deviation (SD)) of the mean frequency of the mechanomyographic and mechanotendographic signals of the triceps brachii muscle (MMGtri) and its tendon (MTGtri) as well as of the abdominal external oblique muscle (MMGobl) during the 15s and fatiguing trials comparing PIMA vs. HIMA. The group M, SD, coefficient of variation (CV) and p-values of statistical comparisons between HIMA and PIMA are displayed. In case of significance, the effect size r is given.

		Mean frequency [Hz]											
		MMGtri				MTGtri				MMGobl			
		15s-trial		fatigue		15s-trial		fatigue		15s-trial		fatigue	
Couples		PIMA	HIMA	PIMA	HIMA	PIMA	HIMA	PIMA	HIMA	PIMA	HIMA	PIMA	HIMA
1	*1*	11.8 ± 0.9	12.9 ± 1.2	11.2 ± 0.6	12.5 ± 1.3	10.8 ± 0.5	13.1 ± 0.9	11.9 ± 0.8	11.8 ± 0.0	12.4 ± 0.5	12.1 ± 0.6	12.4 ± 0.4	11.9 ± 1.2
	2	13.8 ± 0.4	14.2 ± 0.1	14.5 ± 0.3	13.2 ± 0.7	12.0 ± 0.6	13.0 ± 0.8	12.2 ± 1.9	11.3 ± 0.6	14.0 ± 0.9	13.9 ± 0.3	14.4 ± 0.8	11.5 ± 0.6
2	*3*	12.8 ± 0.9	11.9 ± 0.5	13.0 ± 0.3	11.4 ± 0.1	10.7 ± 0.3	10.4 ± 0.5	9.5 ± 0.6	9.6 ± 0.2	13.5 ± 0.7	14.9 ± 0.3	14.0 ± 0.4	14.4 ± 0.5
	4	13.7 ± 0.2	13.4 ± 0.6	14.1 ± 0.4	13.8 ± 0.1	13.5 ± 0.6	14.4 ± 0.4	13.6 ± 0.8	11.5 ± 0.5	18.0 ± 0.5	17.1 ± 1.0	15.1 ± 0.7	16.7 ± 1.1
3	*5*	14.2 ± 0.2	14.0 ± 0.5	13.0 ± 0.3	12.8 ± 0.6	12.2 ± 1.0	11.9 ± 0.9	12.3 ± 0.5	12.3 ± 1.3	13.7 ± 0.8	13.5 ± 0.3	13.3 ± 0.7	13.6 ± 0.7
	6	13.6 ± 0.2	12.8 ± 0.2	13.3 ± 0.1	12.2 ± 0.1	15.0 ± 0.4	13.7 ± 0.8	13.2 ± 0.8	13.2 ± 0.9	16.0 ± 0.2	14.6 ± 0.2	13.4 ± 0.1	15.3 ± 1.2
4	*7*	-	-	-	-	13.4 ± 1.1	14.7 ± 0.1	13.1 ± 0.5	11.5 ±	13.8 ± 0.9	14.1 ± 1.4	14.8 ± 0.8	14.2 ± 1.5
	8	13.3 ± 0.9	13.2 ± 0.3	13.2 ± 0.0	14.5 ± 0.6	12.6 ± 0.9	11.0 ± 1.0	11.7 ± 0.6	13.1 ± 1.3	13.8 ± 0.7	13.7 ± 0.2	13.2 ± 0.1	14.1 ± 0.1
5	*9*	13.9 ± 0.1	14.2 ± 0.4	14.4 ± 0.3	13.5 ± 0.5	12.3 ± 0.7	12.7 ± 0.8	11.7 ± 0.7	11.4 ± 1.5	13.7 ± 0.8	13.1 ± 0.1	12.3 ± 0.0	11.9 ± 0.2
	10	16.2 ± 0.9	16.4 ± 0.7	15.2 ± 0.2	14.6 ± 0.5	12.9 ± 1.4	12.4 ± 0.4	15.1 ± 0.8	12.3 ± 0.5	15.5 ± 0.2	14.4 ± 0.1	13.1 ± 1.3	14.7 ± 0.9
6	*11*	14.3 ± 0.3	13.9 ± 0.9	13.0 ± 0.4	13.3 ± 1.1	-	-	-	-	12.5 ± 0.2	12.3 ± 0.7	13.4 ± 0.1	13.3 ± 0.1
	12	14.2 ± 1.3	15.4 ± 0.6	15.1 ± 0.3	13.6 ± 0.7	-	-	-	-	15.1 ± 0.2	12.3 ± 0.3	16.0 ± 0.8	15.4 ± 1.0
7	*13*	12.8 ± 0.3	12.6 ± 0.3	12.4 ± 0.1	13.2 ± 0.6	13.2 ± 0.4	15.9 ± 0.9	12.2 ± 0.2	14.9 ± 0.9	13.2 ± 0.3	13.7 ± 0.2	13.3 ± 0.1	13.1 ± 0.4
	14	14.1 ± 0.8	14.3 ± 0.4	13.2 ± 0.0	13.6 ± 0.3	13.0 ± 1.4	13.6 ± 0.3	12.1 ± 0.8	12.3 ± 1.1	15.0 ± 0.1	-	14.0 ± 1.0	14.4 ± 0.1
8	*15*	11.9 ± 0.2	13.1 ± 0.3	11.7 ± 0.0	12.6 ± 0.1	11.9 ± 0.4	11.6 ± 0.4	13.1 ± 0.3	9.9 ± 0.8	12.3 ± 0.5	14.0 ± 0.3	12.6 ± 0.1	12.9 ± 0.4
	16	16.1 ± 0.1	14.5 ± 0.4	15.2 ± 0.3	15.1 ± 0.9	13.3 ± 0.5	12.0 ± 1.0	12.5 ± 0.4	12.2 ± 1.4	15.4 ± 0.2	16.0 ± 0.0	15.2 ± 0.3	17.1 ± 0.1
9	*17*	12.3 ± 1.4	12.2 ± 1.1	13.3 ± 0.4	9.9 ± 1.0	12.0 ± 0.1	10.5 ± 0.7	11.1 ± 1.3	9.6 ± 0.3	12.1 ± 0.6	12.9 ± 0.2	12.3 ± 0.7	13.1 ± 0.9
	18	14.5 ± 0.7	14.4 ± 0.4	14.6 ± 2.3	13.6 ± 0.3	12.4 ± 0.8	11.1 ± 1.7	12.1 ± 0.2	10.6 ± 0.6	14.5 ± 0.1	13.8 ± 0.5	13.8 ± 0.1	13.8 ± 0.9
10	*19*	13.9 ± 0.9	13.8 ± 0.6	14.0 ± 0.0	14.8 ± 0.1	11.4 ± 1.0	12.5 ± 0.1	13.1 ± 0.6	10.7 ± 0.4	14.0 ± 0.6	15.2 ± 0.1	13.8 ± 0.4	13.0 ± 0.1
	20	13.7 ± 0.1	11.3 ± 1.4	13.6 ± 0.1	13.3 ± 0.2	11.9 ± 0.8	10.3 ± 1.2	12.5 ± 0.4	12.3 ± 0.4	13.4 ± 1.2	13.1 ± 1.6	13.6 ± 0.3	13.8 ± 0.1
	M	13.737	13.598	13.569	13.234	12.473	12.479	12.377	11.692	14.097	13.929	13.697	13.906
	SD	1.165	1.220	1.120	1.218	1.037	1.578	1.149	1.346	1.443	1.273	1.025	1.462
	CV	0.085	0.090	0.083	0.092	0.083	0.126	0.093	0.115	0.102	0.091	0.075	0.105
	p (r)	0.507		0.229		0.985		0.065		0.631		0.411	
